# Science at the Church Congress

**Published:** 1891-10-17

**Authors:** 


### The Doctor's Armchair.

### SCIENCE AT THE CHURCH CONGRESS.

Mr. Andrew Lang says the world can never become scientific: certain of the speakers at the recent Church Congress are evidently of opinion that the Church can. There need be no hesitation in affirming not only that the Church can, but that she must become scientific if she is to save her soul, and even her body, alive. The Venerable Archdeacon Wilson, better known to many as the Rev. J. M. Wilson, formerly head master of Clifton School, read a paper at Rhyl on "Confronting new Problems," which was as scientific as the heart of a Huxley or of a Darwin could desire. The Archdeacon is a brave man and a religious, and he has not found in his experience of life that religion has tended to make him either less severely honest, or less ardently desirous of exploring things down to their very roots than nature intended him to be. On the contrary, he would say that religion had made him not only morally more sensitive, but also more highly and nobly scientific than he would have been without her.

It will be of service to men of science generally to see how Archdeacon Wilson approaches the critical study of his own religion, the Christian faith, and what interesting results he brings forth. The New Testament is the literary and historical quarry out of which modern Christianity is being dug. "The criticism of the New Testament," says the Archdeacon, "is a complete examination of the documents of which it consists, a discussion of their authorship, date, sources, purpose: the historical value of the statements, and the philosophic value of the opinions therein expressed, with complete liberty to regard them, prior to examination, in the same light as any other literature." This is the fullest possible concession of all that science insists upon. It is in essential accord with what may be called the fundamental principles of human nature. The human mind, if it is conscious of any fundamental fact at all, is conscious of its own freedom. It feels that it possesses the right to question everything, in the heaven above, in the earth beneath, or in the waters under the earth; even to question the Infinite Himself: and it feels its power to give always a discriminating and responsible, though not always a complete and final answer. The human mind, being such as it is, cannot accept, without thought or questioning, any claims that are made upon it which involve the abdication of its freedom or the denial of its discriminating responsibility. No book, however venerable, no institution, however imposing, has the right to say to the meanest sane human being—"Thou shalt, without thought, and on my authority, accept this; or thou shalt, without thought, and on my authority, reject that."

Archdeacon Wilson takes up his position on this scientific standing ground when he approaches the study of the New Testament. Martin Luther, perhaps almost unconsciously, took up precisely the same position when he approached the consideration of the Catholic Church and priesthood. The position is essentially human, and at the same time essentially scientific. It postulates not only that there was a just and reasonable field of enquiry for philosophic mental activity in past ages, but that there is a just and reasonable field of enquiry at the present moment, and for all future time. It breathes the firm hope that as there was a living God who could be discovered, and who could satisfy the minds of those who reverently sought for Him in past ages, so there may be a living God who can be discovered, and the knowledge of whom can satisfy the minds of reverent and intelligent seekers to-day, and in all future time. To take up the opposite attitude, of authority as against enquiry, either on behalf of the Bible or of the Church, is to put a premium on unenquiring scepticism, and to drive all truly scientific minds into open revolt against Christianity, and, perhaps, even against religion itself.

Archdeacon Wilson is a great general who concentrates his forces upon the critical heart of the battle. He says: "It must be regarded as impossible to trace with certainty the origin, relationship, and dates of the Gospels;" but that "the synoptic Gospels probably existed before the close of the first century, and that they truly represent the Apostolic teaching about our Lord, and truly represent the substance and method of His teaching, if not the very words He used." But is not this "substance and method" the very thing that science is always asking for, not only with regard to every book and institution, but with regard to every animal and plant with which she is called upon to deal in any kind of practical way? A zoologist, for example, sees an animal. He begins the important work of classification immediately by noticing its essential characteristics. Has it a backbone and limbs? Then it is a vertebrate animal. Has it breasts like the dog, or an udder like the cow? Then it is a mammalian. Has it two legs instead of four? Then it is a biped as well as a mammal, and it belongs to the order of primates. That is the fundamental fact about it, and the essential character of the creature; and no unessential details, such as colour of hair, shape of head, presence or absence of tail, habits of life, environment, or any other circumstance whatsoever can alter these fundamental facts by a single hair's breadth. The animal is a vertebrate, a mammal, and a primate by every law and fact of the visible and invisible world for ever and ever.

It is precisely thus with the New Testament. The book gives us the actual substance and method of the teaching of Jesus Christ, and it is by that substance and by that method, and by nothing else whatsoever, that the religion of Jesus Christ is to be classified, and to take its place among the religions of the world, and among the moral and the social forces of the world.

What does this scientific criticism of the New Testament practically effect for the educated man and the man of science? Archdeacon Wilson tells us in a sentence, which, however, will be nothing less than a revelation to many inquiring and ingenuous minds. "Criticism," he says, "by emphasising the inadequacy of the record, frees us from the terrible dilemma of accepting what we find incredible, or rejecting the whole as mythical; and it throws us back on the broad historic fact that from Jesus of Nazareth did spring the regeneration of society, and forces us to find in him some explanation of this." Here, again, we have exactly what science asks for, "a broad historic fact." This broad historic fact both invites and insists upon explanation. Those scientific men who, while making it their special function to deal with broad historic facts in the region of morals, elect to pass by this greatest of all modern historic facts—simply confess either their want of courage or their want of capacity to deal with it.

Archdeacon Wilson, with genuine and noble enthusiasm, ventures into the region of prophecy. "Criticism," he says, or rather "the movement towards the recognition of it, may inaugurate a revival of religious feeling." Not only does he foresee such a purifying and morally beneficial prospect ahead, but he is convinced that such a revival will be as timely as the Evangelical movement of the past century or the Anglican deepening of devotion of the present, and probably in its permanent and widespread effects it may be destined to far transcend them both. No scientific man can undervalue public and private morality, and no man of philosophic mind can fail to see that morality which has its root in duty to God, and its development in the environment of a pure Christianity is the most beautiful of all the moral flowers and fruits which the world has yet shown us. If the Church can give us a Christianity which science can honestly accept she will not only ensure her own immortality, but she will confer upon a struggling and sternly fighting world present blessings and future hopes which cannot be valued by any standard but that of the highest known to man.

